# Protocol for conducting bibliometric analysis in biomedicine and related research using CiteSpace and VOSviewer software

**DOI:** 10.1016/j.xpro.2024.103269

**Published:** 2024-09-05

**Authors:** Qiang Du, Rui Zhao, Qianyi Wan, Siyu Li, Huanyu Li, Daofeng Wang, Cheong Wong Ho, Zhihao Dai, Yi Chen, Dan Shan

**Affiliations:** 1 Division of Gastrointestinal Surgery, Department of General Surgery, West China Hospital, Sichuan University, Chengdu, Sichuan, China; 2 Clinical Trial Center, National Medical Products Administration Key Laboratory for Clinical Research and Evaluation of Innovative Drugs, West China Hospital, Sichuan University, Chengdu, Sichuan, China; 3 West China School of Nursing, Sichuan University, Chengdu, Sichuan, China; 4 Department of Pharmacology, School of Pharmacy, China Medical University, Shenyang, Liaoning, China; 5 Liaoning Key Laboratory of Molecular Targeted Anti-Tumor Drug Development and Evaluation, Liaoning Cancer Immune Peptide Drug Engineering Technology Research Center, Key Laboratory of Precision Diagnosis and Treatment of Gastrointestinal Tumors (China Medical University), Ministry of Education; Shenyang, Liaoning, China; 6 Sports Medicine Service, Capital Medical University Affiliated Beijing Jishuitan Hospital, No. 31, Xinjiekou East Street, Beijing, China; 7 Clinical Science Institute, University Hospital Galway, Galway, Ireland; 8 School of Medicine, Royal College of Surgeons, Ireland, University of Medicine and Health Sciences, Dublin, Ireland; 9 Department of Infection Control, West China Hospital, Sichuan University, Chengdu, China

**Keywords:** Bioinformatics, Cell Biology, Health Sciences, Metabolism

## Abstract

Here, we present a protocol for conducting bibliometric analysis in biomedicine using CiteSpace and VOSviewer. We describe the steps for extracting data from Web of Science, data cleaning, and preprocessing. We then detail procedures for identifying research trends and collaboration networks by visualizing data with CiteSpace; mapping co-authorship, co-citation, and keyword co-occurrence using VOSviewer; and analyzing highly cited literature to identify key publications and trends. Finally, we outline techniques for interpreting the visualizations to draw meaningful conclusions about the research landscape.

For complete details on the use and execution of this protocol, please refer to Li et al.^1^

## Highlights

• Verify institutional access to essential databases for comprehensive data retrieval
• Install CiteSpace and VOSviewer for effective bibliometric analysis and visualization
• Retrieve and clean data from WoS to ensure accuracy and consistency in analysis
• Visualize and interpret bibliometric data to spot research trend and key contribution

## Abstract (editor highlights)

Publisher’s note: Undertaking any experimental protocol requires adherence to local institutional guidelines for laboratory safety and ethics.

## Graphical abstract

**Figure:**
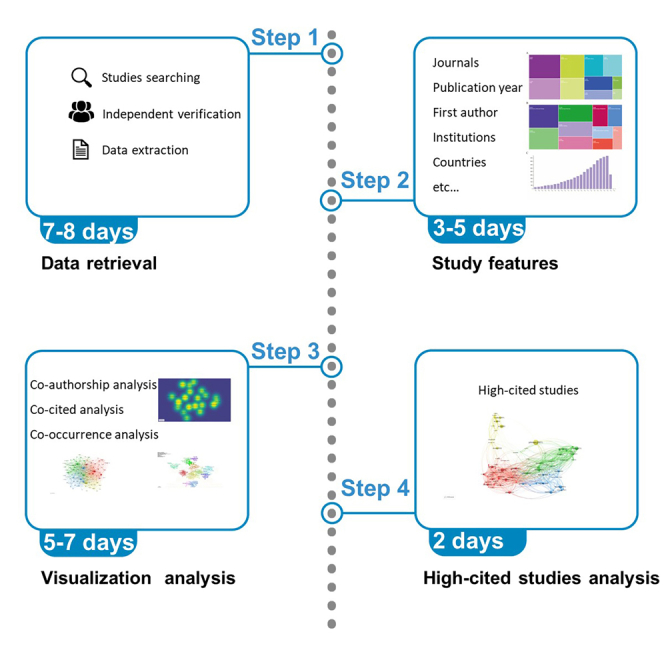

## Before you begin

### Overview

Research in information science is crucial for academic discourse.^2^ This study synthesizes and scrutinizes findings within specific domains, providing a comprehensive evaluation of existing knowledge and consensus.

Analyze connections between scientific studies to uncover new research avenues or refine existing objectives.^3^^,^^4^ Employing bibliometric analysis to enhance the evaluation of research outputs with a clear and systematic approach. This technique emphasizes principal trends, key figures, and pivotal topics, while the visualization of bibliometric data effectively illustrates patterns of scientific collaboration and knowledge dissemination.^5^

Utilize tools like CiteSpace and VOSviewer for effective bibliometric analyses.^4^^,^^6^ Developed by Chaomei Chen, CiteSpace pinpoints and illustrates research focal points by analyzing clusters of publications.^4^ Co-citation analysis identifies the joint citation of references, demonstrating mutual significance and impact.^7^^,^^8^ Keyword co-occurrence analysis highlights key research areas and directions.^9^^,^^10^ VOSviewer constructs and visualizes bibliometric networks, including journals, researchers, and articles based on co-citation, bibliographic coupling, or co-authorship relationships.^11^ It provides graphical representations of scientific landscapes, facilitating visual exploration of interrelationships and collective impacts. Integrate VOSviewer’s visualization capabilities with CiteSpace’s analytical depth for a holistic study view. Use VOSviewer for initial explorations and presentations, while CiteSpace delves into specific trends or clusters. The combined use of VOSviewer and CiteSpace harnesses the strengths of both platforms, providing a powerful toolkit for bibliometric analysis.^12^ Together, they cover a broad spectrum of bibliometric needs, from detailed analytical tasks to high-quality visual presentations, enhancing the comprehensiveness and effectiveness of scholarly research.

This report presents a bibliometric analysis of mitochondrial research using the capabilities of CiteSpace and VOSviewer. This investigation outlines the steps involved in retrieving publications, extracting data, and conducting comprehensive visual analyses. These standardized methods are designed to help researchers efficiently collect relevant publications, extract essential data, and perform in-depth visual analyses. Moreover, this analysis provides new insights by validating earlier trends, identifying influential authors, and uncovering emerging research areas. Specifically, the detailed network visualizations highlight key research clusters and reveal connections between influential studies. This deeper analysis confirms the robustness of initial findings and offers a more nuanced understanding of the research landscape, enabling the identification of pivotal authors and seminal works that shape the field. The primary aim is to standardize bibliometric research methodologies within biomedicine and related fields, thereby enhancing consistency across various research teams.

### Database and software equipment preparation

At present, researchers commonly utilize the Web of Science (WoS), Scopus, PubMed or Google Scholar database for data collection and CiteSpace and VOSviewer software for visualizing knowledge maps. This methodology relies on the capabilities of these tools.

1. Verify that the affiliated institution possesses access to the WoS, Scopus, PubMed or Google Scholar database, particularly the WoS Core Collection.
2. Acquire and install an authorized version of CiteSpace.
3. Install VOSviewer software to complement the analysis and visualization capabilities provided by CiteSpace.

***Note:*** Utilize unlicensed software can result in the loss of crucial node data, which may render the visualization analysis unreliable and unpredictable.

## Key resources table

**Table:** 

REAGENT or RESOURCE	SOURCE	IDENTIFIER
**Software and algorithms**		

CiteSpace	Chen et al.^4^	https://citespace.podia.com/
VOSviewer	Centre for Science and Technology Studies (CWTS)^6^	https://www.vosviewer.com/

**Other**		

Web of Science	N/A	https://webofscience.com/wos
Scopus	N/A	www.scopus.com
PubMed	N/A	https://www.ncbi.nlm.nih.gov/pubmed
Google Scholar	N/A	https://scholar.google.com/

## Step-by-step method details

To demonstrate our focus and objectives, this document utilizes research targeting mitochondrial therapies as a case study to conduct bibliometric research through both CiteSpace and VOSviewer. Table 1 shows the estimated timeframes for each step in the bibliometric analysis.

### Clarify retrieval strategy and inclusion criteria

**Timing: 1 week**

Here, we describe steps for retrieving relevant publications from the WoS database. Table 2 shows the research retrieval strategy used in our bibliometric analysis.

1. Define the research field and keywords.
  a. Use MeSH (Medical Subject Headings) to create subject headings and their corresponding entry terms (free terms).
2. Perform data retrieval in the WoS Core Collection database.
  a. Search for publications in Web of Science using these subject headings and free terms.

***Note:*** WoS does not have a subject heading database; therefore, use the "Title" and "Abstract" fields for retrieval to improve recall and precision of publications.

3. Conduct searches independently.
  a. At least two researchers should independently conduct searches.
  b. Identify and analyze the retrieved articles.
  c. In cases of ambiguity, a third researcher, preferably a senior one, should make the final decision.
4. Conduct searches independently.
  a. At least two researchers should independently conduct searches.
  b. Identify and analyze the retrieved articles.
  c. In cases of ambiguity, a third researcher, preferably a senior one, should make the final decision.
5. Exclude reviews, non-English studies, and conference papers from the analysis.

***Note:*** Because targeted research is highly time-sensitive, we have limited the search period to the past five years.

***Note:*** The search strategy for each database is detailed in the Methods S1.

**Table 1:** Estimated time frames for each step of the bibliometric analysis

Step	Task description	Estimated time frame
**Data retrieval**		

Database Access	Verify access to WoS Core Collection	1 day
Search Query Design	Develop and refine search queries for specific topics	2–3 days
Initial Search	Conduct initial searches to gather relevant literature	1–2 days
Filtering and Refinement	Apply filters and refine search results for relevance	2–3 days
Download Data	Download the filtered search results for analysis	1 day

**Data preparation**		

Data Cleaning	Clean and format data for consistency	2–3 days
Data Integration	Integrate data from multiple sources if necessary	1–2 days

**Visualization**		

Tool Setup	Install and configure CiteSpace and VOSviewer	1 day
Data Import	Import cleaned data into visualization tools	1 day
Preliminary Analysis	Run initial visualizations to check data integrity	1–2 days
Visualization Tuning	Adjust visualization parameters for optimal clarity	2–3 days

**Analysis**		

Interpretation	Analyze visualizations to extract meaningful insights	2–3 days
Reporting	Document findings and insights from visualizations	2–3 days

**Table 2:** Search strategy in Web of Science

Step	Query	Results
#1	Key1= (mitochondria∗ or "mitochondrial function" or "mitochondrial DNA" or "mitochondrial therapy")	
#2	Key2= (Molecular Targeted Therapies) or (Targeted Therapy, Molecular) or (Therapy, Molecular Targeted) or (Targeted Molecular Therapy) or (Molecular Therapy, Targeted) or (Targeted Molecular Therapies) or (Therapy, Targeted Molecular) or (Target∗ Therapy∗) or (Target∗ treatment) or (Target∗ drug∗)	
#3	TI = Key1	200,621
#4	TI = Key2	1,616,496
#5	#3 and #4	2,368
#6	AB = Key1	376,482
#7	AB = Key2	1,609,937
#8	#6 and #7	47,458
#9	#6 and #7 and Article (Document Types)	34,806
#10	#6 and #7 and Article (Document Types) and English (Languages)	34,406
#11	#3 and #4 and #6 and #7 and Article (Document Types) and English (Languages) and Publication Years (2020–2024)	11,721
#12	Two researchers independently check the title and abstract and excluded irrelevant studies (e.g., reviews, comments, conferences, etc.)	11,547

Initial data: Jun 24, 2024; AB: Abstract; Key: Keyword; TI: Title.

### Data extraction based on WoS

**Timing: 2 weeks**

Here, we describe steps regarding the extraction and cleaning of data for analysis.

6. Harness the inherent capabilities of the WoS.
  a. Explore the essential attributes of publications and scrutinize each article.
  b. Extract crucial details such as publication year, authors, institutions, countries, publication journal, journal citation reports, and keywords.
7. Export the raw data and create tables.

**Table 3:** Top 10 most productive countries/regions related to targeting mitochondrial therapies

Country	Number of publications	Total of citations	Average citations
China	5812	65302	11.24
USA	2600	40552	15.60
India	602	4906	8.15
Germany	527	6449	12.24
South Korea	507	5830	11.50
Italy	502	6158	12.27
England	497	6851	13.78
Japan	403	4173	10.35
Spain	308	3942	12.80
France	303	4392	14.50

**Table 4:** Top 10 most productive institutions related to targeting mitochondrial therapies

Institution	Number of publications	Total of citations	Average citations
Sun Yat-Sen University	274	3625	13.23
Shanghai Jiao Tong University	267	3386	12.68
Chinese Academy of Sciences	255	4128	16.19
Fudan University	249	3377	13.56
Zhejiang University	228	3098	13.59
Nanjing Medical University	198	2074	10.47
Southern Medical University	188	1796	9.55
Sichuan University	180	2308	12.82
Central South University	172	1903	11.06
Huazhong University of Science and Technology	167	2650	15.87

**Table 5:** Top 10 most productive authors related to targeting mitochondrial therapies

Author	Number of publications	Total of citations	Average citations
Wang, Wei	47	728	15.49
Liu, Yang	44	464	10.55
Zhang, Wei	43	571	13.28
Li, Yan	43	477	11.09
Yang, Yang	38	797	20.97
Li, Wei	35	396	11.31
Zhang, Jie	35	352	10.06
Liu, Yi	34	605	17.79
Zhang, Hao	33	303	9.18
Wang, Yi	29	514	17.72

**Table 6:** Top 10 most productive journals related to targeting mitochondrial therapies

Journal	Number of publications	Total of citations	Average citations
International Journal of Molecular Sciences	399	2573	6.45
Cancers	182	1427	7.84
Scientific Reports	178	1467	8.24
Cell Death & Disease	165	2408	14.59
Free Radical Biology and Medicine	140	2175	15.54
Nature Communications	139	3885	27.95
Redox Biology	138	3114	22.57
Frontiers In Pharmacology	132	1445	10.95
Antioxidants	120	795	6.63
Cells	116	787	6.78

***Note:*** All basic characteristics of publications can be obtained in WoS based on the top 10 most productive countries/regions, institutions, authors, and journals (refer to Tables 3, 4, 5, and 6).

**Figure 1:**
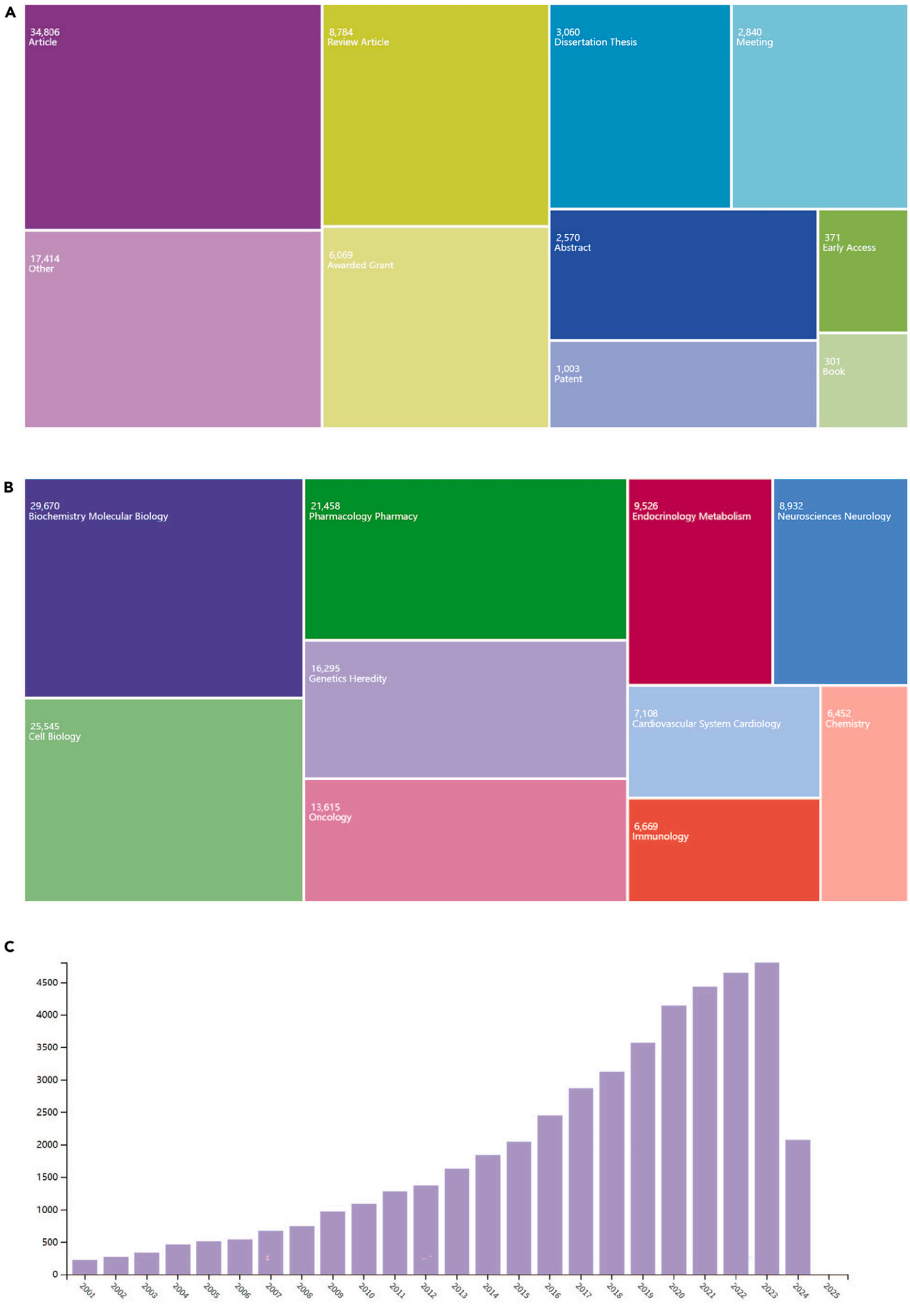
The extraction of publication-related information details from Web of Science (A) Article type; (B) Research field; (C) Annual publication count.
  b. Export data.

***Note:*** Use professional plotting software to enhance the presentation of data visualization results.

8. Utilize the “Analyze Results” function on WoS.
  a. View data such as publication years, authors, publication titles, countries/regions, and other journal-related information (see Figure 1).

9. Export all publications in “plain text” format.

***Note:*** This format only supports exporting 500 publications at a time. If the search results include more than 500 publications, perform multiple exports. Name the exported file in the format “download_xxx-xxx”.

**CRITICAL:** In bibliometric analysis, the selection of a comprehensive and reliable database is crucial. In this protocol, we have chosen the WoS. Compared to other databases, WoS offers several advantages.

First, WoS provides a broad and deep coverage of scientific literature across multiple disciplines. Unlike PubMed, which focuses primarily on biomedical literature, and Scopus, which has a wider scope but less depth in certain areas, WoS includes a balanced mix of sciences, social sciences, arts, and humanities.

Second, WoS is renowned for its citation indexing, which allows for comprehensive citation analysis. This feature is less developed in PubMed, which primarily serves as a repository for abstracts and some full-text articles without extensive citation tracking.

Third, the rigorous selection criteria for journals included in WoS ensure high data quality and reliability. This makes WoS a preferred choice for conducting bibliometric analyses that require accurate and credible data.

These features make WoS the most suitable database for conducting a thorough and reliable bibliometric analysis in biomedicine and related research.

### Visualization analysis based on VOSviewer

**Timing: 1 week**

For visualizing the analysis results, VOSviewer can be utilized. Here is a specific tutorial on how to use VOSviewer for data visualization.

10. Download and Install VOSviewer.
  a. Go to the VOSviewer website (www.vosviewer.com/download).
  b. Download the latest version.
  c. Install the software following the provided instructions.
11. Prepare Data for VOSviewer.

***Note:*** Ensure your data is in a format compatible with VOSviewer. Typically, it’s a plain text file exported from WoS.

12. Import Data into VOSviewer.
  a. Open VOSviewer.
  b. Select “Create a map based on text data” if you are visualizing keywords, or “Create a map based on bibliographic data” for citation information.
  c. Follow the prompts to upload your plain text file.
13. Configure Visualization Settings.
  a. Adjust the settings according to your needs.

**Figure 2:**
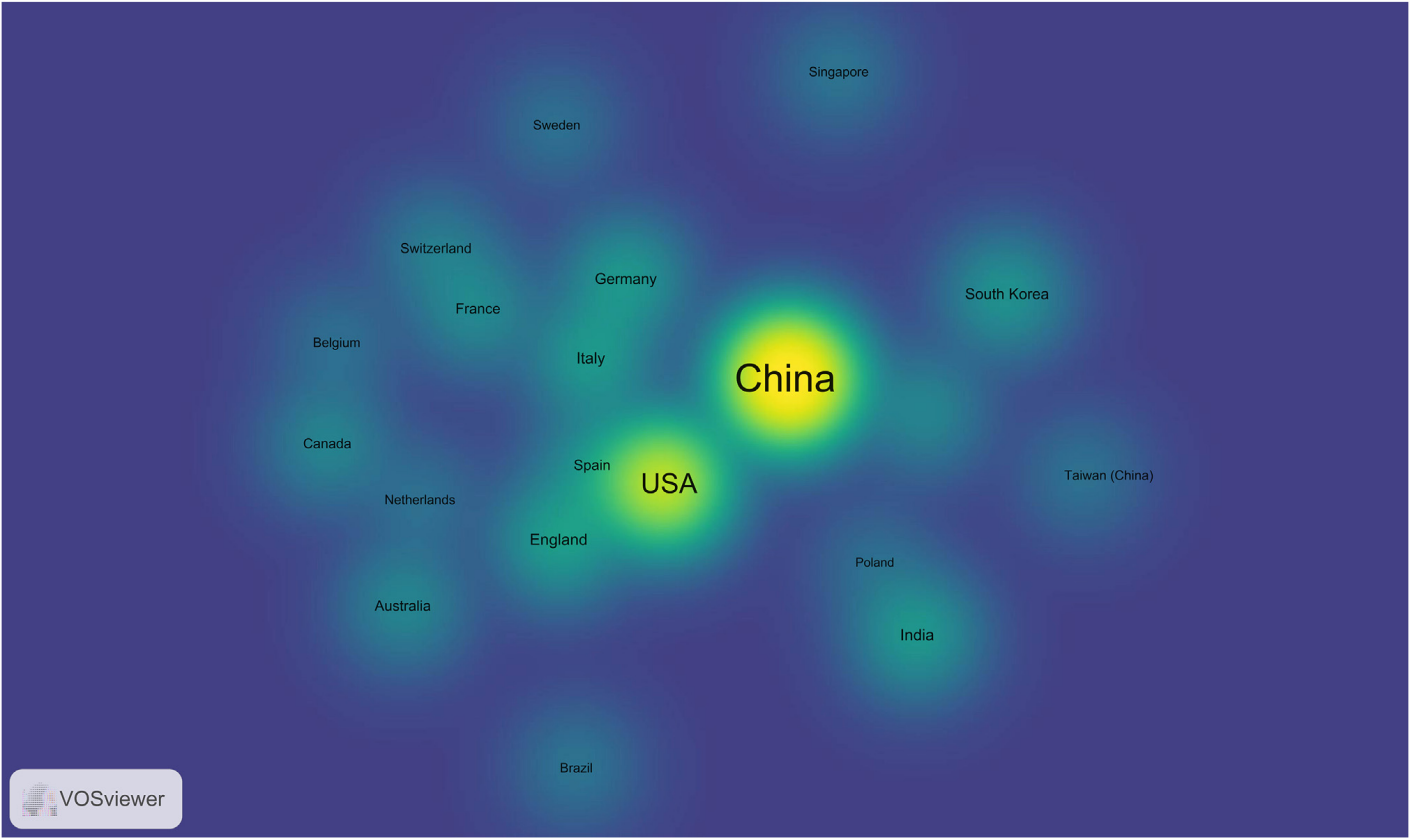
Visualization mapping and clusters of the most productive countries/regions related to targeting mitochondrial therapies

**Figure 3:**
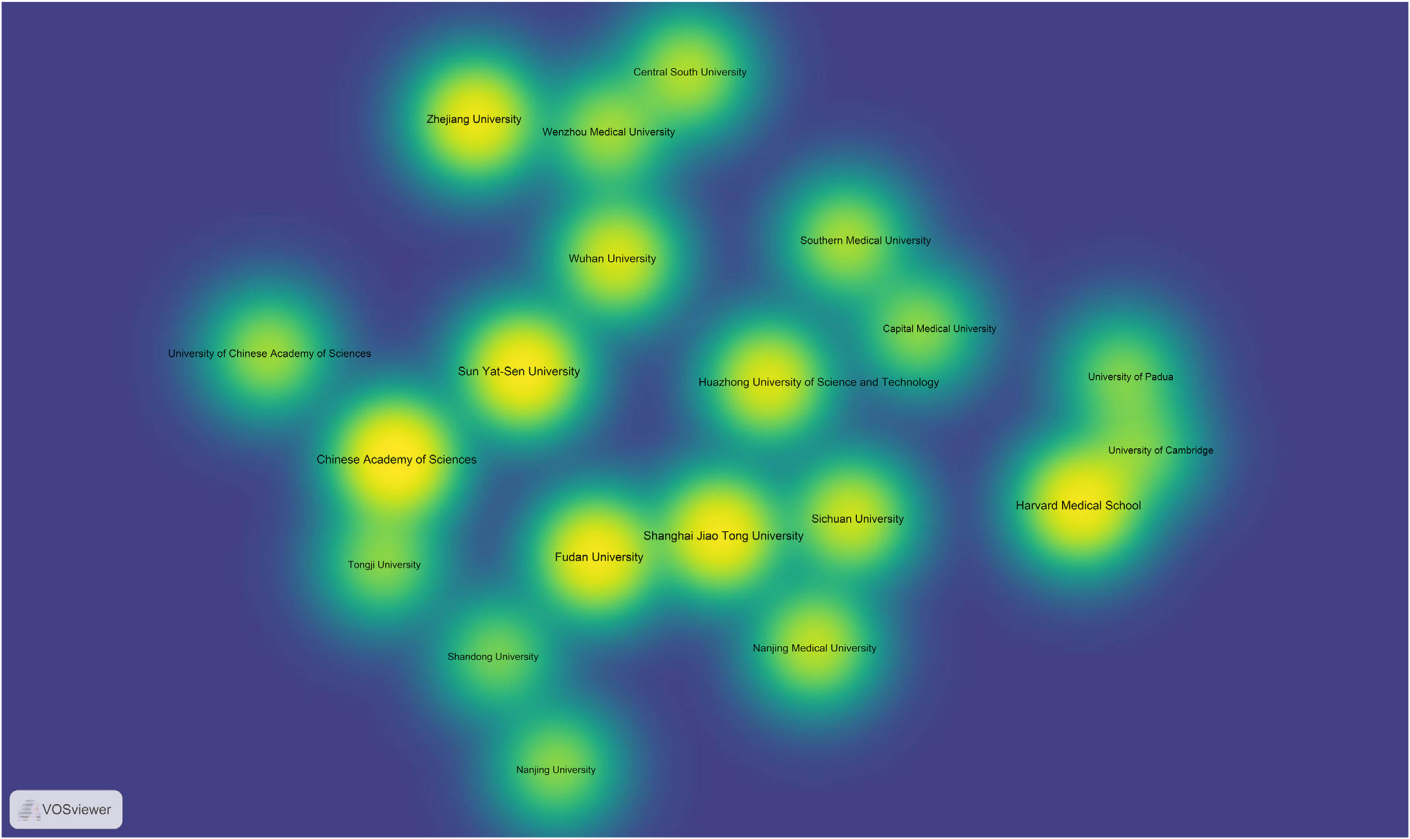
Visualization mapping and clusters of the most productive institutions related to targeting mitochondrial therapies

**Figure 4:**
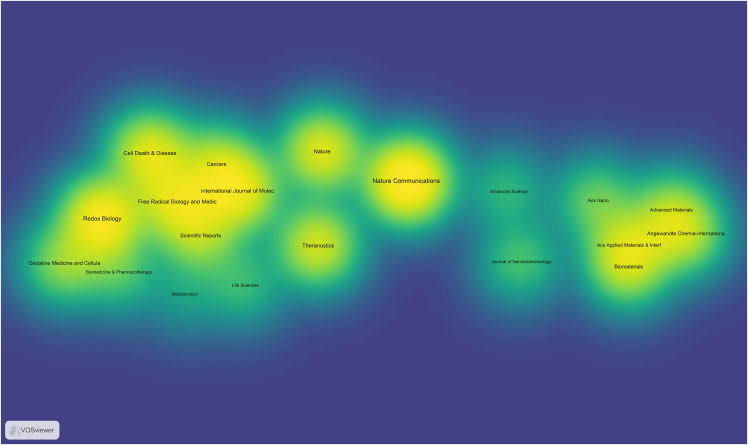
Visualization mapping and clusters of the most productive journals related to targeting mitochondrial therapies

***Note:*** VOSviewer can process the data and display the map, showing relationships and clusters within your data (Figures 2, 3, and 4).

14. Generate the Map.
  a. Click on the “Create” button to generate the visualization.

***Note:*** You can choose different types of maps (e.g., countries/regions, institutions, and journals) and customize the visualization parameters.

**Figure 5:**
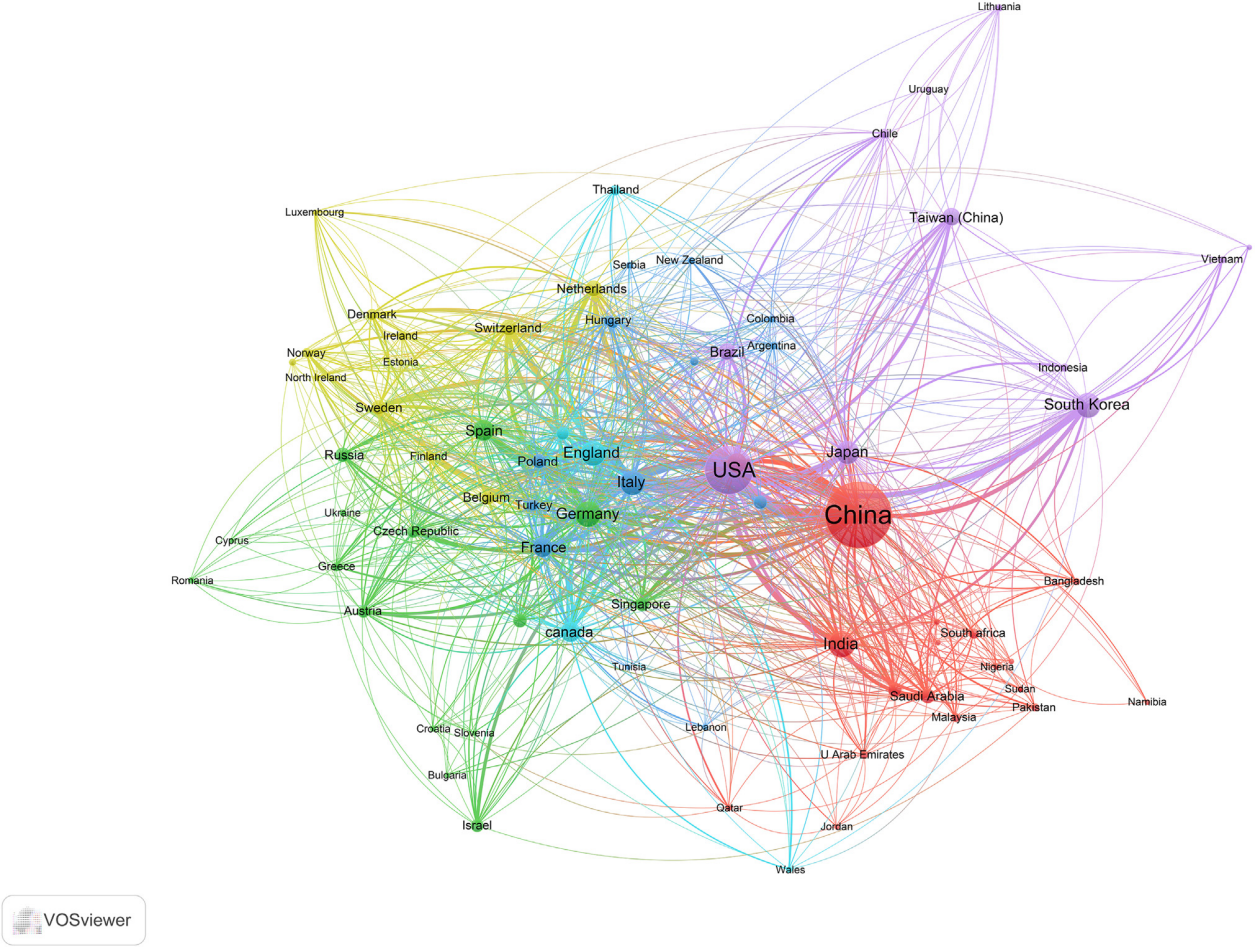
Collaborative network analysis of countries/regions involved in targeting mitochondrial therapy research

**Figure 6:**
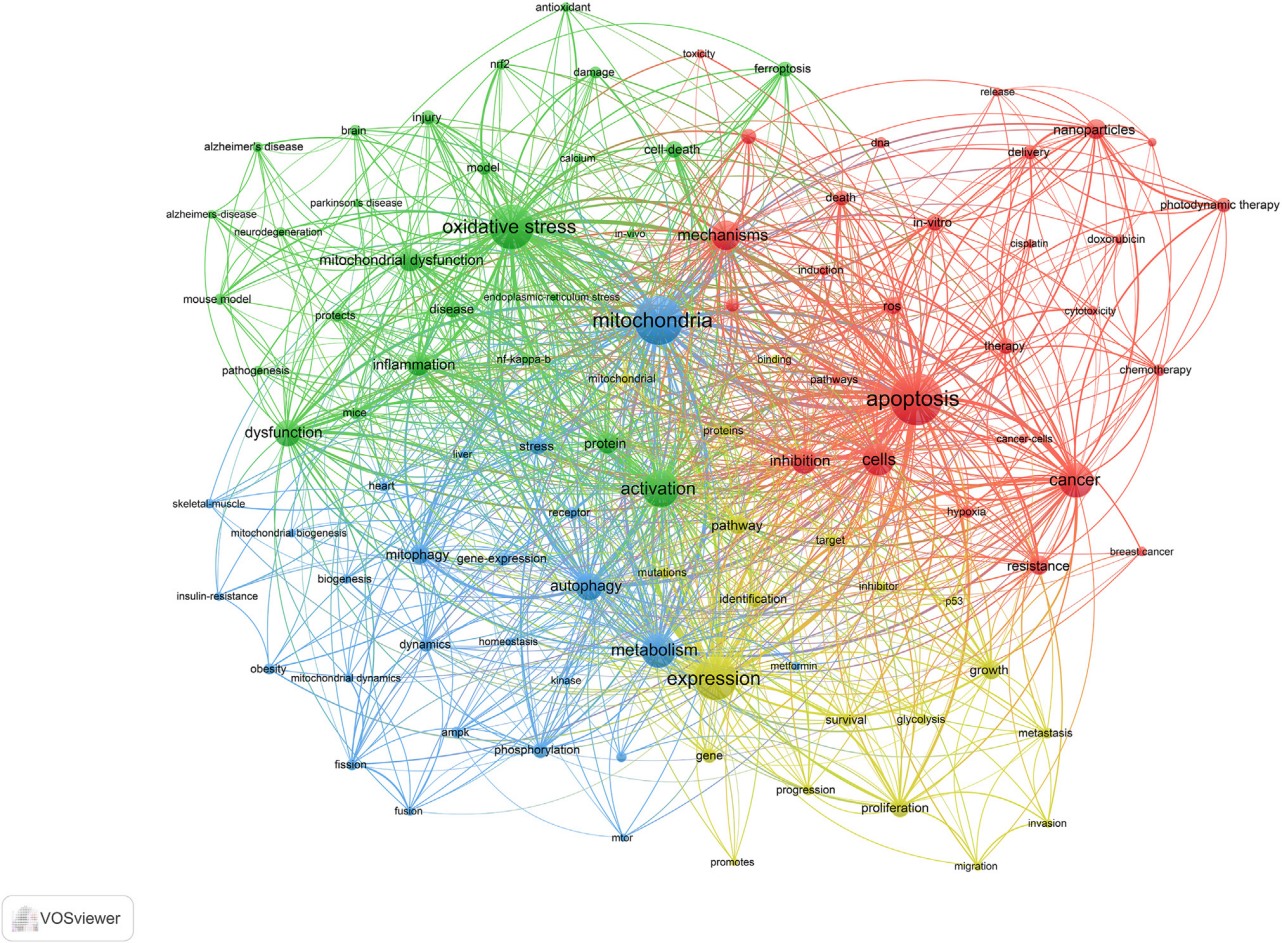
Collaborative network analysis of research keywords involved in targeting mitochondrial therapy research

**Figure 7:**
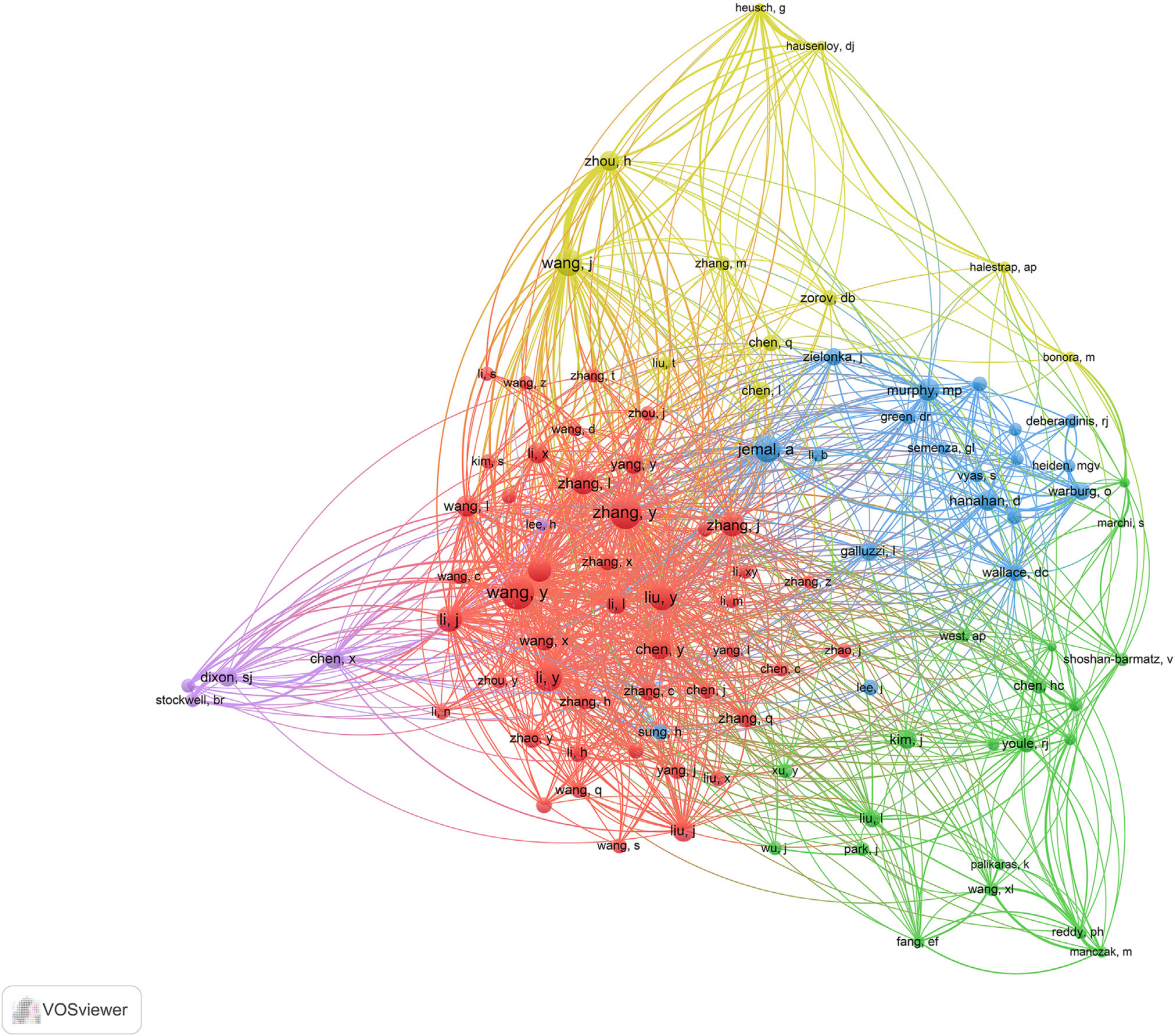
Collaborative network analysis of authors involved in targeting mitochondrial therapy research

***Note:*** By following this tutorial, you can effectively visualize and analyze your research data using VOSviewer, enhancing your ability to present complex information clearly and comprehensively.

15. Analyze and Interpret the Map.
  a. Use the interactive features of VOSviewer to zoom in on clusters.
  b. Explore connections between items.
  c. Interpret the patterns displayed.
16. Export the Visualization.
  a. Export the satisfied visualization in your preferred format (Figures 5, 6, and 7).

### Visualization analysis based on CiteSpace

**Timing: 1 week**

Here, we describe the steps for visualizing bibliometric data using CiteSpace.

17. Download and Install CiteSpace.
  a. Go to the CiteSpace website (https://citespace.podia.com/download).
  b. Download the Basic version.
  c. Configure the corresponding Java runtime environment.
18. Prepare Data for CiteSpace.
  a. Process the exported file into a format recognized by CiteSpace.
  b. Create three folders named “data”, “project”, and “input”.
  c. Store the downloaded files from WoS in the “input” folder.
  d. Use CiteSpace to read and convert them into a recognizable format.
  e. Export them to the “data” folder.
19. Create a New Analysis Module.
  a. Ensure the software reads the “data” folder smoothly.
  b. Set the time slice to 1 in the workspace.
  c. Leave all parameters at their default values except for those in the Node Types module.

***Note:*** If the visualization results are unsatisfactory, use the Pruning option and adjust node distances for better dispersion.

20. Keyword Co-occurrence Analysis.
  a. Select the “Keywords” module.
  b. Run the software.
  c. Perform co-occurrence analysis.

***Note:*** This analysis, also known as keyword analysis, clusters keywords to help identify focal points in a research field.

**Figure 8:**
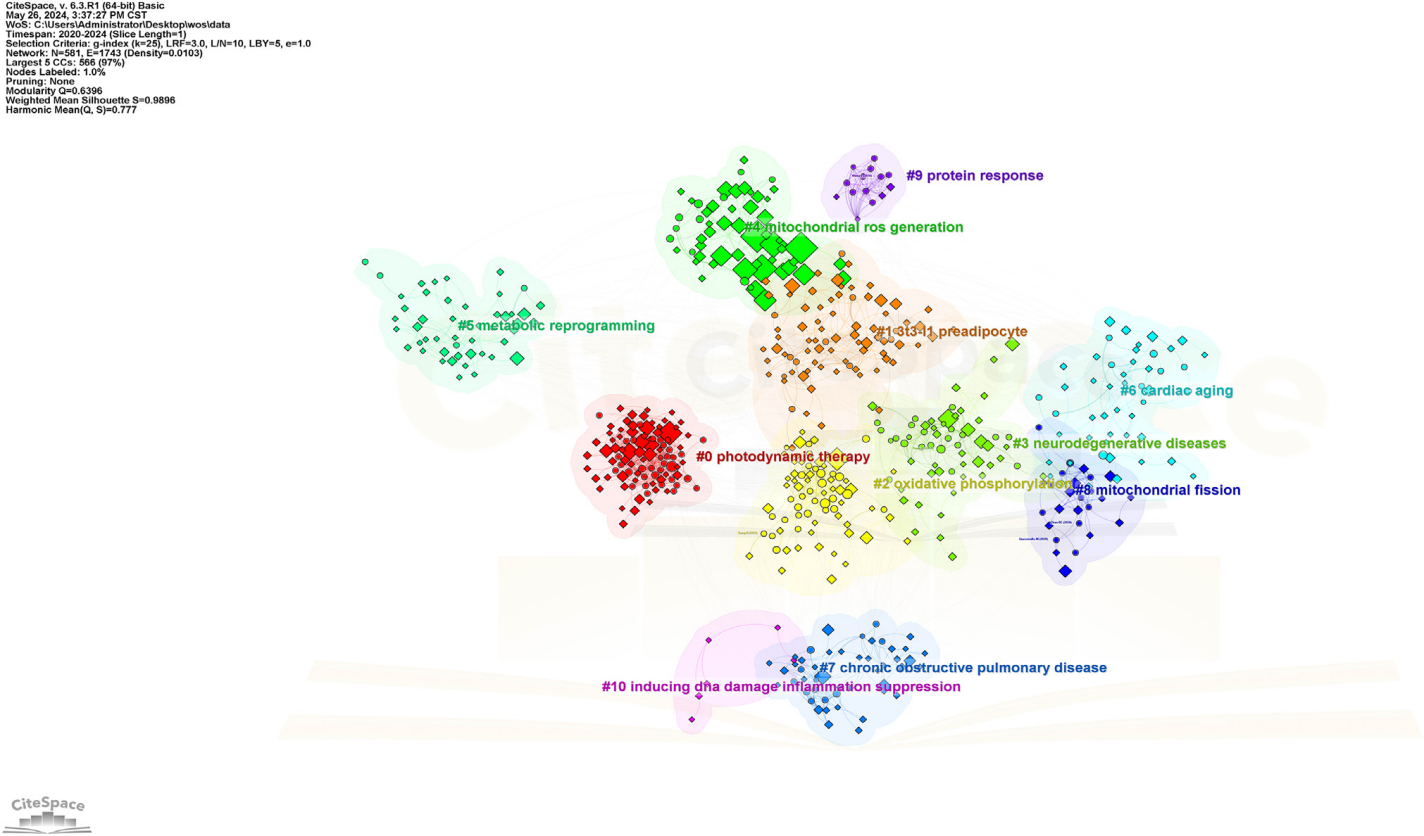
Visualization of keyword co-occurrence analysis by the CiteSpace
23. Authorship and Keyword Co-occurrence Analysis.
  a. Select the “Author” and “Keywords” modules.
  b. Run the software.
  c. Perform co-occurrence analysis.

***Note:*** This co-occurrence analysis reflects the connections between authors and keywords.

21. Adjust Visualization.
  a. Drag the nodes to adjust the visualization results.
22. Cluster Analysis and Export.
  a. Perform cluster analysis.
  b. Export the clustering results.
  c. Integrate the visualization results with the clustering labels (Figure 8).

24. Adjust Visualization.
  a. Drag the nodes to adjust the visualization results.
25. Cluster Analysis.
  a. Perform cluster analysis.
  b. Export the clustering results.
  c. Integrate the visualization results with the clustering labels.

**Figure 9:**
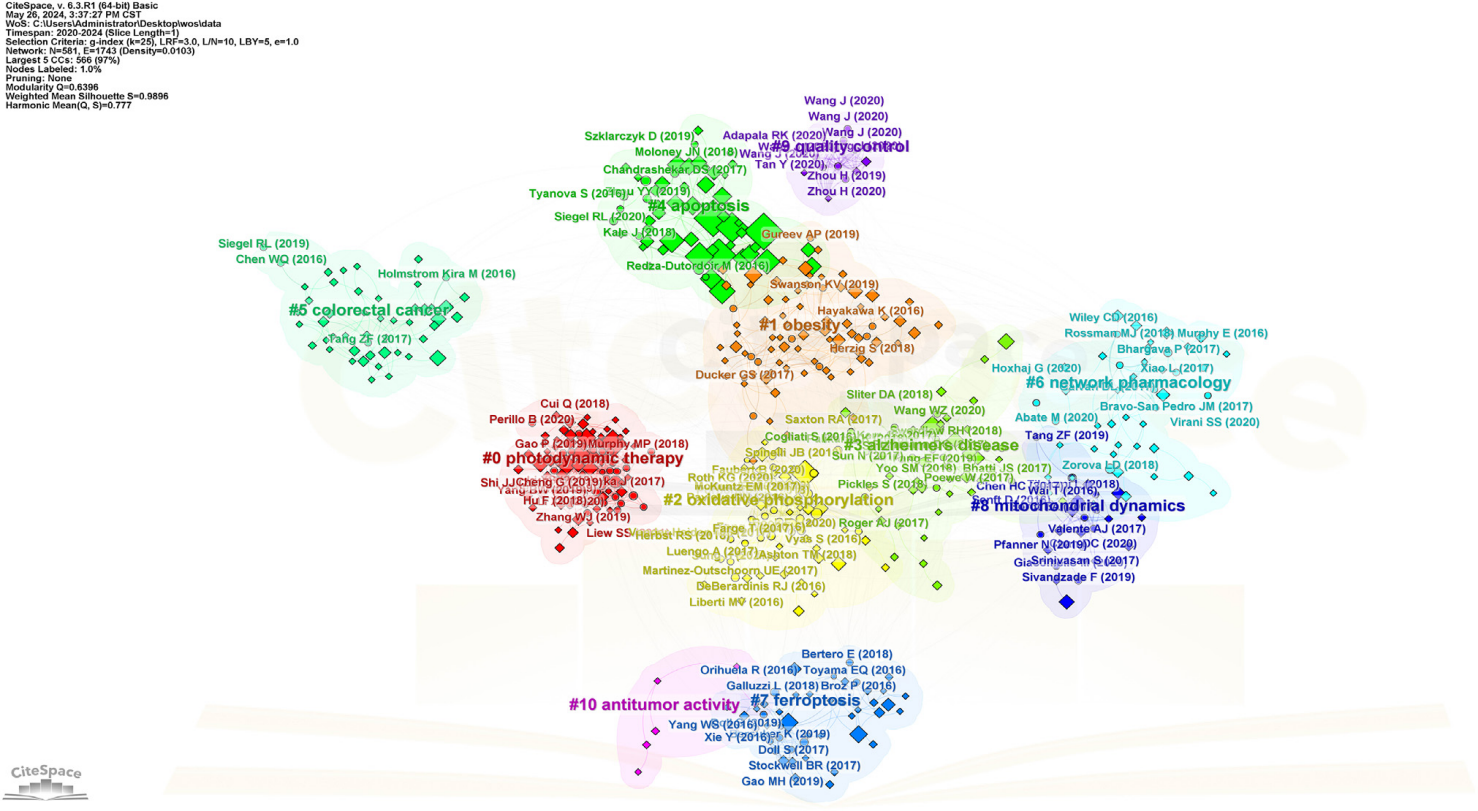
Visualization of authorship and keyword co-occurrence analysis by the CiteSpace

***Note:*** The clustering results and labels can be represented in different colors (Figure 9).

***Note:*** By following these steps, you can effectively use CiteSpace to visualize and analyze your research data, enhancing your ability to present complex information clearly and comprehensively.

### Interpreting visualizations in CiteSpace and VOSviewer

**Timing: 1 week**

Interpreting the visualizations generated by bibliometric tools like CiteSpace and VOSviewer is crucial for drawing meaningful conclusions from the data. This section provides a comprehensive guide on understanding these visualizations, using Figures 6 and 8 as examples.

26. CiteSpace Visualizations.
  a. Nodes and Links.
    i. Nodes: Represent entities such as authors, documents, or terms.
    ii. Links: Indicate relationships between nodes, such as co-authorship, co-citation, or keyword co-occurrence.
  b. Node Size and Color.
    i. Size: Larger nodes typically indicate higher significance or frequency (e.g., more citations, more publications).
    ii. Color: Different colors represent different time slices or clusters. The progression from warm to cool colors (e.g., red to blue) often indicates the temporal evolution of the research field.
  c. Clusters.
    i. Modularity: Clusters are identified using modularity, which measures the density of links within clusters compared to links between clusters. High modularity indicates well-defined clusters.
    ii. Labeling: Clusters are often labeled with terms that best represent the core theme of the cluster. These labels help in understanding the primary topics of research.
  d. Centrality: Nodes with high centrality (often marked with purple rings) are critical in connecting different clusters, indicating their importance in the network.
    ***Note:*** Example Analysis of Figure 8:
    Figure 8 represents a timeline view of the co-citation network, showing the evolution of research themes over time. Each node signifies a cited reference, and their distribution along the timeline reflects the development of research interests.
    Evolution of Research: The timeline reveals the progression of key research areas. For instance, the emergence of terms related to “photodynamic therapy” and “mitochondrial ROS generation” in recent years indicates growing interest in these innovative treatment strategies.
    Influential Works: Highly cited references, represented by larger nodes, highlight seminal works that have significantly influenced the field. These pivotal studies form the backbone of the current understanding and guide future research directions.
    Emerging Themes: The clustering of newer nodes in specific areas suggests emerging themes in the literature. For example, the concentration of recent nodes around “metabolic reprogramming” and “neurodegenerative diseases” indicates burgeoning research interest in these topics, possibly driven by advances in related technologies or methodologies.
27. VOSviewer Visualizations.
  a. Network Visualization.
    i. Nodes: Represent entities like keywords, authors, or documents.
    ii. Links: Indicate relationships such as co-authorship or co-occurrence.
    iii. Node Size and Color: Similar to CiteSpace, with larger nodes indicating higher frequency or impact and colors representing different clusters or time periods.
  b. Density Visualization: Color Gradients: Indicate the density of items in a particular area. Warmer colors (e.g., red, yellow) indicate higher density, suggesting areas of intense research activity.
  c. Overlay Visualization: Temporal Information: Nodes are colored based on temporal data, showing the evolution of research topics over time.
    ***Note:*** Example Analysis of Figure 6:
    The network visualization map in Figure 6 illustrates the co-occurrence of keywords in the field. Each node represents a keyword, and the links between them indicate the co-occurrence of these terms within the same publications. The color-coding indicates different clusters of related keywords, which can be interpreted as distinct research themes.
    Cluster Analysis: The figure reveals several prominent clusters. For instance, the red cluster highlights research focused on “apoptosis” and “cancer,” indicating a significant volume of studies investigating the role of apoptosis in cancer. The green cluster, centered around “oxidative stress” and “mitochondrial dysfunction,” suggests a strong research interest in the interplay between oxidative stress and mitochondrial health.
    Central Keywords: Keywords like “mitochondria,” “oxidative stress,” and “apoptosis” appear as central nodes with numerous connections, signifying their pivotal role in the research landscape. These terms are frequently studied topics, reflecting their importance in current research trends.
    Research Gaps and Trends: The visualization helps identify research gaps. For example, keywords with fewer connections may represent emerging research areas that have not yet been extensively explored. Additionally, the dense interconnections among certain keywords suggest well-established research fields with rich literature.

### High-cited publication analysis

**Timing: 1 week**

Here, we describe the steps for analyzing highly cited publications to identify significant contributions.

28. Select Highly Cited Publications.
  a. Identify highly cited publications from the search results in WoS.

***Note:*** Web of Science defines articles with prominent citation frequencies as highly cited. These extensively referenced publications are crucial in their respective fields, offering critical insights into research patterns and significant breakthroughs. Highly cited publications are defined as those in the top 10% of citations within the selected time window (2020–2024).

**Figure 10:**
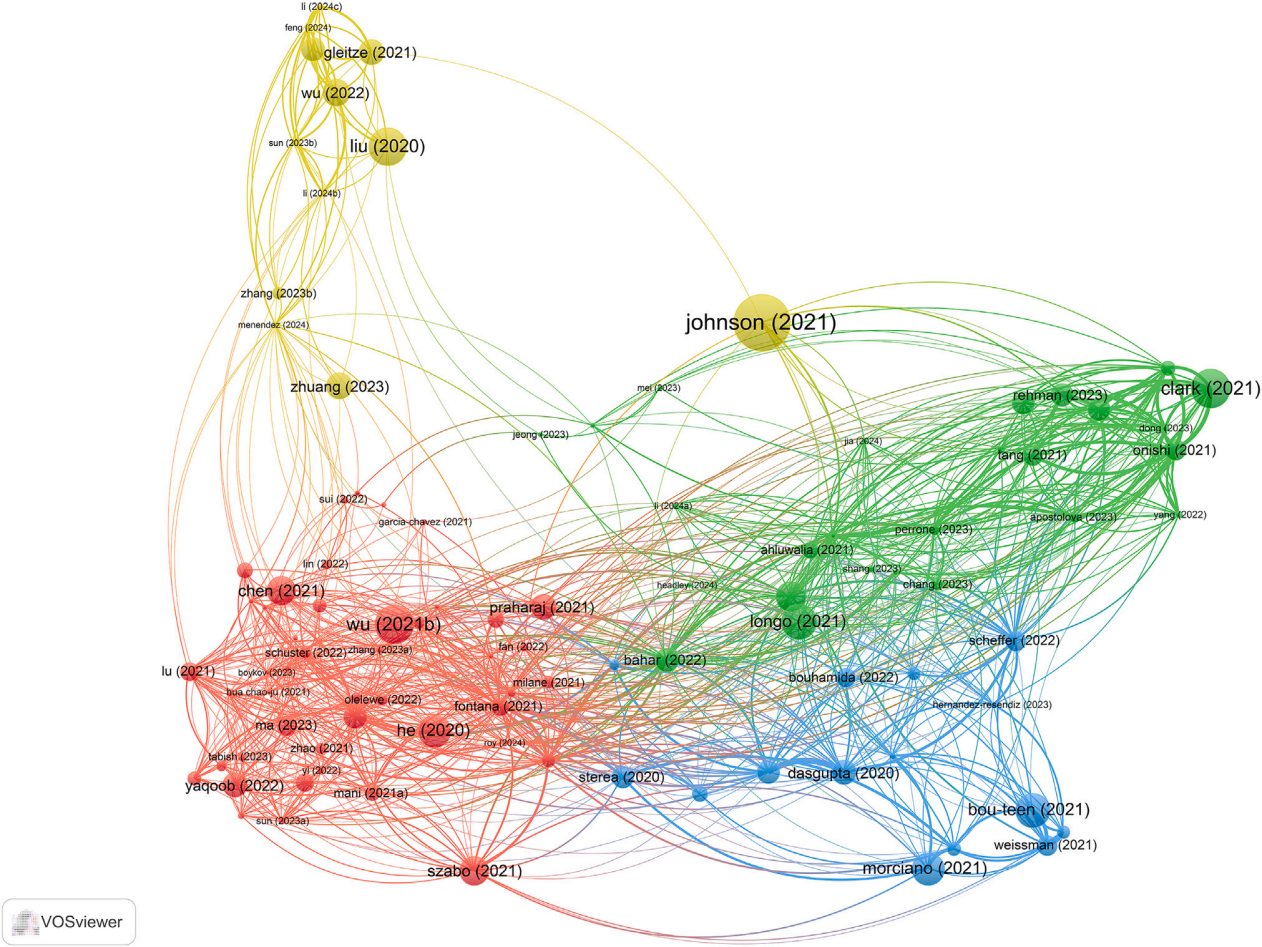
Collaborative network analysis of the top 50 authors involved in targeting mitochondrial therapy research

***Note:*** By following these steps, you can gain a deeper understanding of significant contributions and trends within your field of study.

29. Abstract Review and Data Extraction.
  a. Read the abstracts of highly cited articles individually.
  b. Extract key information such as author and year of publication, etc.
30. Repeat Visualization Analysis.
  a. Perform the visualization analysis on the highly cited articles, following the steps outlined previously (Figure 10).

## Expected outcomes

Adhering to this bibliometric protocol in biomedical science research, researchers can achieve the following results. First, ensure a high recall and precision rate in retrieving relevant publications. Second, accurately extract key features from research literature for clear presentation. Third, create an optimal scientific knowledge graph that visualizes clusters indicating research focal points. Fourth, identify future research directions through detailed analysis of highly cited publications. Following these steps will enhance the effectiveness and clarity of your biomedical research analysis.

## Limitations

This protocol has limitations. It assumes that the framework of scientific knowledge can be represented through text-based semantic similarities or explicit citation links, which requires validation within specific disciplines to ensure its effectiveness. Additionally, this protocol relies exclusively on the WoS database, which may introduce bias by favoring the contents of this particular repository. Moreover, CiteSpace and VOSviewer rely on complex algorithms whose parameter settings, such as time slicing, node types, similarity measures, and resolution, can introduce biases and affect the visualization and interpretation of results. Finally, while bibliometric analysis is a powerful tool for understanding research trends and patterns, several inherent limitations and potential biases need to be considered such as publication bias, keyword search bias and citation analysis limitations.

## Troubleshooting

### Problem 1: Irrelevant publication retrieval

When searching for publications, a significant amount of unrelated research literature is often collected.

### Potential solution: Enhancing search specificity

To address the absence of MeSH in WoS, using a targeted search that combines the "Title" and "Abstract" fields can significantly reduce the inclusion of unrelated literature. It is also recommended for 2–3 researchers to independently conduct the search and compare their results. Publications should be ranked by relevance, with careful evaluation of the least relevant ones before inclusion. See step 1.

### Problem 2: Inconsistency in VOSviewer visualization

VOSviewer visualization results can sometimes be inconsistent.

### Potential solution: Enhancing VOSviewer visualization consistency

VOSviewer may not always produce consistent visualization results due to variations in its internal algorithms. To address this issue, users can adjust the visualization settings after generating the initial map. Fine-tuning parameters such as resolution, clustering techniques, and node positioning can help achieve more reliable and consistent results. Additionally, conducting multiple iterations of the visualization process and comparing the outcomes can further enhance the accuracy and stability of the visualizations. This approach ensures a more robust and dependable analysis. See step 3.

### Problem 3: Inconsistency in CiteSpace visualization

CiteSpace visualization results can sometimes be inconsistent.

### Potential solution: Acknowledging CiteSpace limitations

CiteSpace may not always reproduce visualization results reliably due to its internal algorithms. To address this, users can perform cluster analyses after visualization, adjust the number and arrangement of nodes, and then finalize the visualization. This approach is both user-friendly and efficient. See step 4.

### Problem 4: Redundancy in visualization analysis

Duplicate institutional names and keywords can appear in visualization analyses.

### Potential solution: Data optimization

To maintain a clear and concise data set, inspect the initial output table and remove duplicate keywords and institutions after executing the visualization command. This helps ensure accuracy in subsequent analyses. See step 3 and step 4.

## Resource availability

### Lead contact

Dan Shan: D.Shan6@universityofgalway.ie.

### Technical contact

Qiang Du: duqiangokk@163.com; Rui Zhao: zhaorui0122@126.com; Qianyi Wan: 2759067085@qq.com; Yi Chen: toddy@scu.edu.cn.

### Materials availability

This study did not generate new unique reagents.

### Data and code availability

The data and code obtained from our study are available from the corresponding author on reasonable request.

## Acknowledgments

This study was supported by the Health Research Board (HRB) in Ireland (grant reference: SS-2023-054) and the 1·3·5 project for disciplines of excellence—Clinical Research Incubation Project, West China Hospital, Sichuan University (grant reference: ZYAI24024).

## Author contributions

The study conception was contributed by Q.D., R.Z., and Q.W., while D.S. and Y.C. played a significant role in the study design. All authors were involved in material preparation, data collection, and analysis. Q.D., R.Z., and Q.W. took responsibility for writing the manuscript, while D.S. and Y.C. carried out the paper’s revision and polishing.

## Declaration of interests

The authors declare no competing interests.
