# Supplementary material for: Protocol for conducting bibliometric analysis in biomedicine and related research using CiteSpace and VOSviewer software
Source: STAR Protoc. 2024 Sep 5;5(3):103269. doi: 10.1016/j.xpro.2024.103269 (PMC11408372; doi:10.1016/j.xpro.2024.103269)
Supplement: Document S1. Methods S1 [file mmc1.pdf]

Methods S1: search strategy for each database (i.e., Web of Science, PubMed, Scopus, and Google Scholar), related to Step 1.

■ Web of Science (WoS)

Search Strategy:

(TI=(mitochondria* OR "mitochondrial function" OR "mitochondrial DNA" OR "mitochondrial therapy") AND TI=("Molecular Targeted Therapies" OR "Targeted Therapy, Molecular" OR "Therapy, Molecular Targeted" OR "Targeted Molecular Therapy" OR "Molecular Therapy, Targeted" OR "Targeted Molecular Therapies" OR "Therapy, Targeted Molecular" OR "Targeted Therap*" OR "Targeted treatment" OR "Targeted drug*")) AND (DT=Article) AND (LA=English) AND (PY=2020-2024)

■ PubMed

Search Strategy:

((("Mitochondria"[MeSH Terms] OR "mitochondria"[All Fields] OR "mitochondrial function"[All Fields] OR "mitochondrial DNA"[All Fields] OR "mitochondrial therapy"[All Fields])) AND (("Molecular Targeted Therapy"[MeSH Terms] OR "Targeted Therapy, Molecular"[All Fields] OR "Therapy, Molecular Targeted"[All Fields] OR "Targeted Molecular Therapy"[All Fields] OR "Molecular Therapy, Targeted"[All Fields] OR "Targeted Molecular Therapies"[All Fields] OR "Therapy, Targeted Molecular"[All Fields] OR "Targeted Therap*"[All Fields] OR "Targeted treatment"[All Fields] OR "Targeted drug*"[All Fields]))) AND (2020/01/01 : 2024/12/31[Date - Publication]) AND (english[Language]) AND (journal article[Publication Type])

■ Scopus

Search Strategy:

TITLE-ABS-KEY(mitochondria* OR "mitochondrial function" OR "mitochondrial DNA" OR "mitochondrial therapy") AND TITLE-ABS-KEY("Molecular Targeted Therapies" OR "Targeted Therapy, Molecular" OR "Therapy, Molecular Targeted" OR "Targeted Molecular Therapy" OR "Molecular Therapy, Targeted" OR "Targeted Molecular Therapies" OR "Therapy, Targeted Molecular" OR "Targeted Therap*" OR "Targeted treatment" OR "Targeted drug*") AND (DOCTYPE(ar)) AND (LIMIT-TO(LANGUAGE, "English")) AND (PUBYEAR > 2019 AND PUBYEAR < 2025)

■ Google Scholar

Search Strategy:

("mitochondria" OR "mitochondrial function" OR "mitochondrial DNA" OR "mitochondrial therapy") AND ("Molecular Targeted Therapies" OR "Targeted Therapy, Molecular" OR "Therapy, Molecular Targeted" OR "Targeted Molecular Therapy" OR "Molecular Therapy, Targeted" OR "Targeted Molecular Therapies" OR "Therapy, Targeted Molecular" OR "Targeted Therap*" OR "Targeted treatment" OR "Targeted drug*") AND (2020..2024) AND (English)
